# Multiscale assessment of habitat selection and avoidance of sympatric carnivores by the endangered ocelot

**DOI:** 10.1038/s41598-023-35271-9

**Published:** 2023-06-01

**Authors:** Maksim Sergeyev, Michael J. Cherry, Evan P. Tanner, Jason V. Lombardi, Michael E. Tewes, Tyler A. Campbell

**Affiliations:** 1 Caesar Kleberg Wildlife Research Institute, Texas A&M University Kingsville, 205 Howe Agricultural Bldg, Kingsville, TX 78363 USA; 2 East Foundation, San Antonio, TX 78216 USA

**Keywords:** Community ecology, Conservation biology

## Abstract

Habitat selection by animals is a complex, dynamic process that can vary across spatial and temporal scales. Understanding habitat selection is a vital component of managing endangered species. Ocelots (*Leopardus pardalis*), a medium-sized endangered felid, overlap in their northern range with bobcats (*Lynx rufus*) and coyotes (*Canis latrans*), with all three species sharing similar space and resource use. As the potential for competition between these three carnivores is high, understanding differences in habitat use and the effect of these potential competitors on habitat selection of ocelots is essential to conservation. Our objective was to compare habitat selection between species and examine if ocelots avoided areas used by competitors at broad and fine scales. We captured and collared 8 ocelots, 13 bobcats, and 5 coyotes on the East Foundation’s El Sauz Ranch and the Yturria San Francisco Ranch in South Texas, USA from 2017 to 2021. We compared 2nd (position of home range) and 3rd (use within the home range) order selection across species and examined whether ocelots avoided areas categorized as high probability of use by bobcats and coyotes across both orders of selection. We found a preference for heterogeneous landscapes by bobcats and coyotes while ocelots were strongly tied to woody cover across both orders. At the 2nd order, ocelots selected areas with higher probability of use by bobcats and showed no response to higher probability of use by coyotes, suggesting ocelots did not avoid either species. However, at the 3rd order, ocelots avoided areas used by coyotes. Ocelots selected for areas of use by bobcats at the 2nd order and 3rd order. Results suggest that at the broader scale, placement of the home range is not affected by the presence of sympatric carnivores, however, at a finer scale, ocelots are avoiding coyotes but not bobcats. Our study emphasizes the importance of woody and herbaceous cover at the broad scale and dense vegetation at the finer scale to sustain ocelots. In addition, we show differing patterns of interspecific avoidance by ocelots across species and scales.

## Introduction

Resource availability is a driving force in shaping animal populations^1^ and as such, understanding resource selection is a central concept within ecology. Selection of resources is defined as a disproportionate use of available conditions and resources^1,2^. Resource selection by animals is shaped by a variety of behavioral processes influenced by various tradeoffs and constraints^3^ and is often a dynamic process that can vary annually, seasonally, or hourly as availability of resources changes^4^. A variety of factors may influence resource selection of animals, such as proximity to water sources, presence of conspecifics or competitors, and the cover types available in the area^5–7^. These processes can vary with spatial scale, a concept known as transmutability^8–10^. Therefore, understanding variation in resource availability and habitat selection across scales is an essential aspect of managing wildlife species.

Habitat selection of animals has been described as scale-dependent, occurring hierarchically from broad to fine-scale selection^1,2,8^. At the broadest scale, 1st order selection describes selection of a geographic range by a species or population, followed by 2nd order selection which describes the placement of a home range on a landscape, 3rd order selection describes use within the home range, and finally 4th order selection, describes use of specific resource patches^1,11^. Scale, in the context of resource selection, is a concept that has been considered in ecology for decades^9,12^, however, with recent advances in wildlife monitoring and remote sensing techniques, our ability to analyze ecological relationships across multiple scales has vastly improved. Many recent studies have observed differences in habitat selection of a species across scales and emphasize the importance of multiscale comparisons^2,3,13–15^. Because spatial scale is of such importance to assessing habitat selection, multiscale assessments provide a more complete understanding of the processes shaping selection of a species and can be of vital importance to assessing the selection of endangered species^1^.

The ocelot (*Leopardus pardalis*) is a medium-sized wild felid, ranging from Texas in the United States to northern Argentina^16^. Ocelots are a species of Least Concern globally, but are listed as endangered in the United States and Mexico, and Vulnerable in Colombia, Brazil, and Argentina^17,18^. Across their expansive geographic range, ocelots are associated with mixed and dense vegetation communities (e.g. dry thorn forest, tropical broadleaf, pine-oak woodlands, tropical deciduous forests)^19–23^. In South Texas, ocelots have been described as heavily dependent on woody cover^21,24–26^. Availability of woody cover has declined within coastal southern Texas due to increased road networks, agricultural and urban development, which has contributed to isolation and loss of connectivity between known populations^27^, leading to the species being listed as federally endangered in the United States^28^. Further, future projections of land cover distributions in the region suggest continued increase of anthropogenic development and severe declines in woody cover^29^. As such, understanding landscape characteristics that best supports ocelots is essential to conserving populations within this area. To this point, however, the majority of studies on ocelots within South Texas have relied on radio telemetry data^24,26,27,30,31^ or camera traps^29,32^. A fine-scale analysis of habitat selection using high-frequency GPS locations of ocelots, conducted at the 2nd and 3rd orders of selection, has not been performed before our study and therefore has the potential to inform recovery strategies and concurrent reintroduction efforts.

The effect of competitor species on the habitat selection of ocelots remains unknown. In Central and South America, medium and small carnivores exhibit spatial avoidance and temporal segregation in response to top-down pressure from sympatric ocelots^33,34^. Other studies in South America found no negative effects of top-down pressure from dominant predators on ocelots^35–37^. In South Texas, however, ocelots are only sympatric with two functionally similar carnivores of comparable size, the bobcat (*Lynx rufus*) and the coyote (*Canis latrans*)^32^. Bobcats and coyotes are often considered habitat generalist species in comparison to ocelots, using a wider array of land cover types^26,38,39^. Despite this, these species extensively co-occur within this area^32^, suggesting a possibility for interspecific competition for space. Further, bobcats and coyotes have extensive overlap in diet and habitat use^38–41^, similar to overlap between bobcats and ocelots^26,42^, suggesting the potential for exploitative competition for resources between these three carnivores. Because of similarity in diet, body size, and space use, these species may compete for resources such as food and space. As populations of ocelots are endangered, interspecific competition for limited resources due to drought or other factors could further affect viability of ocelots in the region. While habitat use of each species has been described in South Texas and other areas, no study has compared habitat selection between these three species, to our knowledge. As these species may have antagonistic interactions, understanding how habitat selection differs and identifying potential competition is a key component of conservation of current ocelot populations and the potential reintroduction of new populations. Prior approaches to describing antagonistic relationships (such as predator–prey dynamics) have involved using the probability of use of a predator species as a predictor variable in models of habitat selection or survival of prey^43–46^. In a similar approach, we consider the presence of potential competitor species in a habitat selection model to assess avoidance between species and examine this process across scales of selection. A comparison across scale allows us to examine avoidance in two different ecological contexts. Avoidance at the broader scale may be the result of differences in habitat requirements while avoidance at a finer scale may reflect interspecific competition for spatial resources, or similarly be a function of niche partitioning.

As a species of conservation concern, understanding how competition shapes the habitat selection of ocelots is of paramount importance to management^1^. Our objectives were to describe the habitat selection of ocelots, bobcats, and coyotes at two orders of selection (2nd and 3rd). Further, we examined whether ocelots avoided areas of higher use by bobcats and coyotes, at two orders of selection. If exploitative competition for space is occurring between ocelots, bobcats and coyotes, then we would expect to see avoidance of areas used by potential competitor species. We predicted that (1) ocelots would exhibit a preference for dense vegetation cover while bobcats and coyotes would use a greater variety of cover types and (2) ocelots would avoid areas used by bobcats and coyotes at both the 2nd and 3rd order. A cross-scale description of habitat selection can provide guidelines for habitat management at both the broader, landscape-scale and at a finer scale within specific areas that sustain ocelots. Understanding the effect of sympatric carnivores on the habitat selection of ocelots is a vital piece in identifying areas for reintroduction of ocelots for conservation efforts.

## Study area

**Figure 1:**
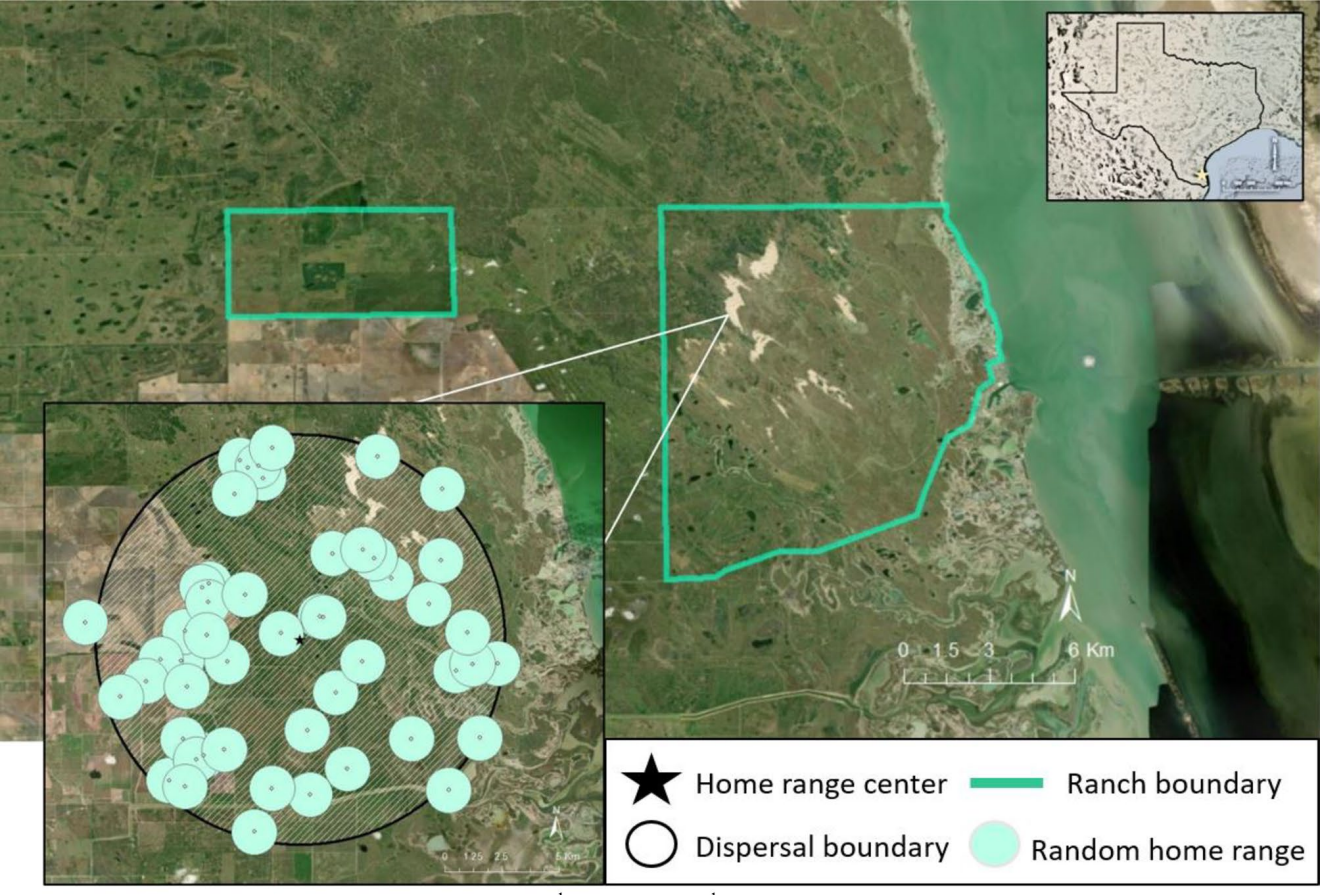
Study area for assessing 2nd order and 3rd order selection of bobcats (*Lynx rufus*), coyotes (*Canis latrans*) and ocelots (*Leopardus pardalis*) in South Texas USA, from 2017 to 2021. Polygons denote ranch boundaries where animals were captured (left = Yturria San Francisco Ranch, right = East Foundation’s El Sauz Ranch). Bottom left portion shows an example of generating 2nd order random home ranges for one individual. Black star designates centroid of the coyote’s true home range, shaded circle represents the buffer distance equal to the dispersal of coyotes (9 km; Hibler^52^), and the smaller circles represent the random home ranges generated, with centroids designated by white points. Background satellite imagery provides context on landscape configuration; light beige represents sand dunes and bare ground, lighter greens are open pastures and grasslands and darker greens show woody vegetation. Figure was created in ESRI ArcMap 10.8 (www.esri.com).

We assessed habitat selection of ocelots, bobcats, and coyotes on the East Foundation’s El Sauz Ranch and the Yturria San Francisco Ranch located in Willacy and Kenedy counties in southern Texas, USA (Fig. 1). El Sauz Ranch (113 km^2^) is a cattle (*Bos taurus indicus*) ranch that prioritizes land stewardship and manages land for cattle and native wildlife. A variety of landscape features are found on the ranch, including coastal estuarine wetlands, grasslands, dunes, artificial water features, prairies, and areas of woody vegetation cover^21^. The Yturria Family’s San Francisco Ranch (25.9 km^2^) emphasizes land stewardship, conservation of ocelots, and hunting of ungulates^31^. Two conservation easements (1.98 km^2^) consisting of highly dense woody vegetation that are owned by the US Fish and Wildlife Service Lower Rio Grande Valley National Wildlife Refuge Complex are located on the ranch^28^. Surrounding patches of restored native woody vegetation are managed by The Nature Conservancy. Vegetation communities on these ranches include larger patches of live oak (*Quercus virginiana*), palm (*Sabal* spp.), and honey mesquite (*Neltuma glandulosa*) forest with thornshrub and herbaceous understories, emergent wetlands with thornshrub patches, and cordgrass (*Spartina* spp.)-thornshrub patches^30^. Woody vegetation species composition includes huisache (*Acacia farnesiana*), snake-eyes (*Phaulothamnus spinescens*), lime prickly ash (*Zanthoxylum fagara*), whitebrush (*Aloysia gratissima*), lotebush (*Ziziphus obtusifolia*), desert olive (*Forestiera angustifolia*), crucifixion thorn (*Castela emoryi*), crucita (*Chromolaena odorata*), and spiny hackberry (*Celtis pallida*)^21,24,31^. This region has a subtropical and semi-arid climate; annual temperatures typically range from 10 °C to 36 °C^47^. Annual rainfall in the region is approximately 68 cm, however, rainfall in the region is highly variable, resulting in episodic droughts^21,28,47^.

## Methods

### Animal capture

We captured 6 ocelots (2 male, 4 female), 12 bobcats (6 male, 6 female), and 5 coyotes (2 male, 3 female) on the El Sauz Ranch and 2 ocelots (1 male, 1 female) and 1 male bobcat on the Yturria Family’s San Francisco Ranch from January 2017 to May 2021. All captured individuals were adults. Animals were captured using single-door Tomahawk box traps (108 × 55 × 40 cm: Tomahawk Trap Co., Tomahawk, WI, USA). We set up to 20 stations of 1–2 box traps each across one to two trap lines in mixed and dense thornshrub and live oak forest on each property from November to May each trapping season. The number of traps varied throughout each season; dependent on weather and personnel availability. We baited the box traps with a live chicken (*Gallus gallus*), or pigeon (*Columbia livia*) separately contained within a compartment inaccessible from the trap^30,31^. We immobilized captured animals using a 4:1 mixture of tiletamine hydrochloride and zolazepam hydrochloride (Telazol™, Zoetis, Florham Park, NJ, USA) at a dose of 5 mg/kg in 2017 and used a mixture of ketamine hydrochloride (4–5 mg/kg) and medetomidine HCl (0.05 mg/kg) and a reversal of 5 mg of atipamezole per 1 mg medetomidine (ZooPharm, Laramie, WY, USA) from 2019 to 2021 (no captures were conducted in 2018)^30,31,48^. Sedation protocols were changed following recommendations from our collaborating wildlife veterinarians. Each captured individual was fitted with a Lotek Minitrack and Litetrack global positioning system (GPS) satellite collar (Lotek™, New Market, ON, Canada). Collars were programmed to record locations every 30–60 min and to automatically drop after either 4–6 months or 1 year; longer fix schedules and drop-off times varied based on a concurrent study using these data. All capture and handling of wildlife were conducted following United States Fish and Wildlife Service permit (#PRT-676811), Texas Parks and Wildlife Department permit (#SP0190-600), and Texas A&M University Kingsville Institutional Animal Care and Use Committee protocol (2015-12-20B, 2019-2-28, 2020-8-28) and all methods were conducted in accordance with the relevant guidelines and regulations as well as in accordance with ARRIVE guidelines.

### 2nd order habitat selection

We estimated the home range of each collared individual using a 95% adaptive kernel density estimate (aKDE). To evaluate selection at the 2nd order, we compared the placement of the true home range with 50 random home ranges (Design III)^49^. We calculated the centroid of each aKDE and simulated the centroid of a random home range within the average dispersal distance for each species (Fig. 1). Dispersal distances used for this step were 7.7 km for male ocelots and 2.5 km for female ocelots^50^, 5.58 km for bobcats^51^, and 9.0 km for coyotes^52^. Dispersal distances of ocelots and bobcats were reported from South Texas. Dispersal of coyotes in South Texas has not been documented, to our knowledge, and the value used above was recorded in Utah (western United States). As no sex-specific values for dispersal were provided for bobcats and coyotes in South Texas, we elected to use the dispersal distance for both males and females. The centroid of each random home range was then buffered based on the size of the observed home range for each individual, resulting in 50 random, circular home ranges equal in area to the observed home range. Within each observed and random home range, we sampled locations at a rate of 100 points/km^2^ to maintain consistent sampling density across individuals and species.

We used unsupervised classified 2018 Landsat 8 imagery (30 m) developed by Lombardi et al.^30^ for the study area to evaluate the influence of landscape structure of woody, bare ground (bare) and herbaceous cover types. Classified imagery (91.9% accuracy) was categorized into six land cover types: woody, herbaceous, agriculture, bare, urban development and water. Landscape metrics were then calculated using an 8-cell (Queen’s rule) moving window analysis with a window size of 116 m (based on the average step length of ocelots) using FragStats 4.4^30,53^. We identified five metrics previously associated with habitat selection of the target species throughout their range^21,30,54^. For bare, herbaceous, and woody cover, we generated values of mean patch area (MPA; ha), landscape shape index (LSI [the ratio of the actual landscape edge length to the minimum possible edge length]), edge density (ED; m/ha), patch density (PD; patches/100 ha), and percent land cover (PLAND; %), totaling 15 variables. We calculated each raster at a 30 m resolution. All variables in the model were standardized by standard deviation.

We modeled habitat selection using mixed-effect logistic regression models with animal ID as a random intercept using the ‘lme4’ package in program R^55^. We evaluated a set of 12 candidate models using an AIC_c_ model selection process^56,57^ and considered models ∆AIC_c_ < 2.0 as competing models. Models were created to represent differing hypotheses on the importance of cover type, fragmentation, and patch size while avoiding combinations with highly correlated variables (cor > 0.6). Top models, according to AIC_c_, for bobcats and coyotes were used to predict the probability of bobcat and coyote use across the landscape. These variables were then included in models of habitat selection for ocelots, to examine whether ocelots were avoiding bobcats and/or coyotes. To avoid issues with multicollinearity, we modified our list of candidate models to not include the probability of bobcats and/or coyotes in the same model with highly correlated variables and evaluated a list of 10 candidate models. Using the output of one model as a predictor variable in another model does not propagate uncertainty from one model to the next, however, we considered this information as useful to describe ocelot behavior and examine avoidance between species.

### 3rd order habitat selection

We examined selection at the 3rd order, within each individual’s home range. We maintained the same estimate of each individual’s home range as used in the 2nd order comparison (95% aKDE). Selection at the second order was evaluated within a bounding area based on dispersal distance and position of random home ranges at a distance from the original, while third order selection was evaluated within each animals home range, thereby resulting in a smaller area examined. Within each home range, we randomly sampled 10 locations for each true location collected. At the third order, the variables considered were vegetation density (vegetation points/cell), percent canopy cover (%), distance (m) to open areas (< 25% canopy cover), distance (m) to dense cover (> 75% canopy cover), and patch shape (the ratio of the actual patch perimeter to the minimum possible perimeter) and area (m^2^) of dense cover^31^. Vegetation density and canopy cover were obtained from light detection and ranging (LiDAR) data collected by the United States Geological Survey for South Texas in 2018^58^ at 70 cm resolution. We classified LiDAR data and calculated landscape metrics in program LP360 (GeoCue, Madison, AL, USA). We calculated distance to open areas, distance to dense cover, and patch size and shape of dense cover from the LiDAR canopy cover raster. We calculated each raster at a resolution of 10 m. All variables in the model were scaled by standard deviation and centered. We evaluated selection using mixed-effect logistic regression models with animal ID as a random intercept. We evaluated a set of 12 candidate models using an AICc model selection and considered models ∆AIC_c_ < 2.0 as competing models. These models represented differing hypotheses on the importance of vegetation structure and landscape composition while avoiding combinations of highly correlated variables (cor > 0.6). We calculated probability of bobcat and coyote use based on the top models based on AICc. These variables were included in the habitat selection models of ocelots. We also included 2nd order probability of bobcats and coyotes to examine if there was any avoidance occurring across scales of selection. To avoid issues with multicollinearity, we modified our list of candidate models for ocelots and evaluated a set of eight candidate models that included probability of bobcats and coyotes at the 2nd and 3rd order, distance to dense cover, vegetation density and patch area.

## Results

**Figure 2:**
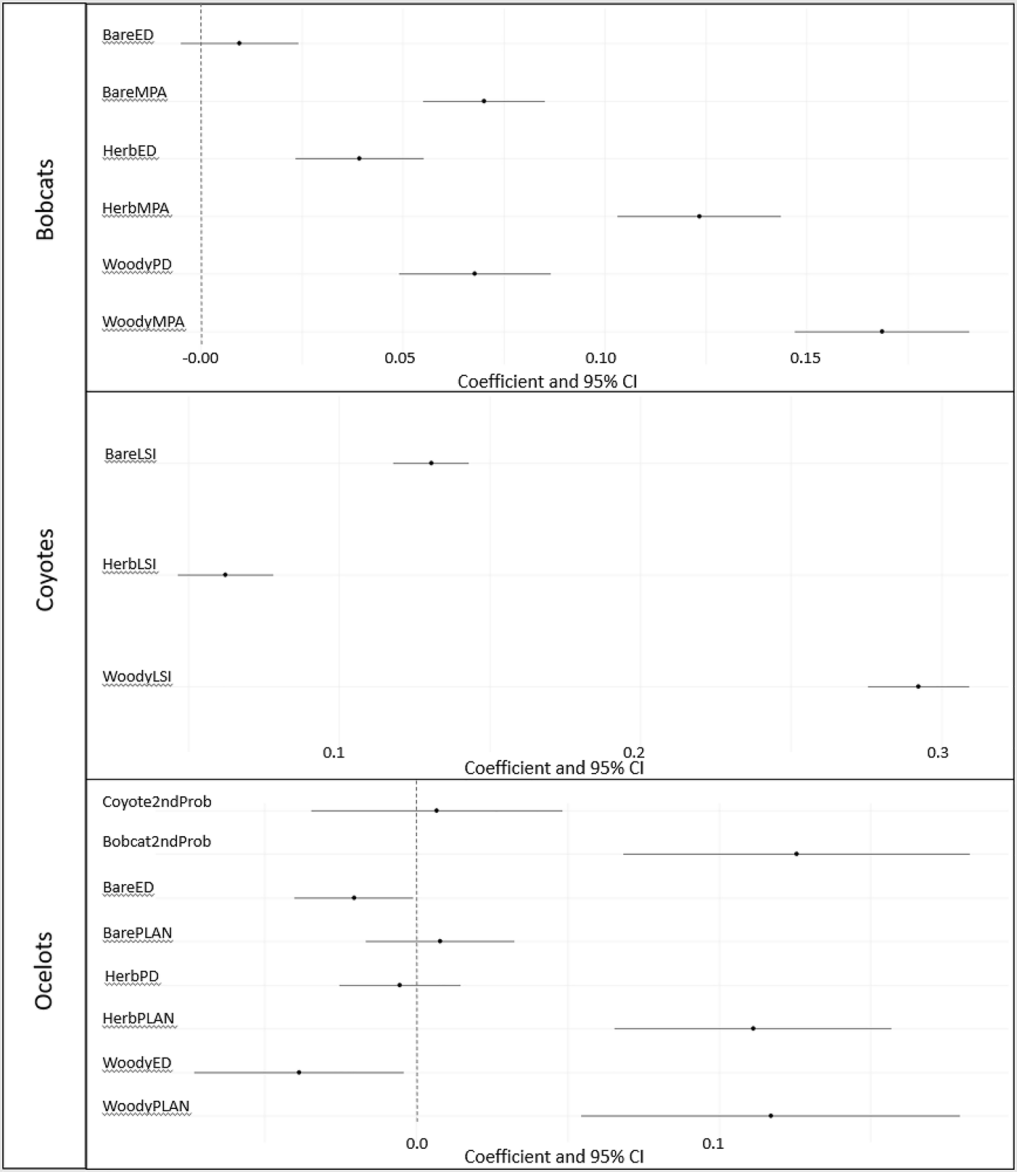
Model coefficients from most supported models describing the 2nd order habitat selection of bobcats (*Lynx rufus*), coyotes (*Canis latrans*) and ocelots (*Leopardus pardalis*) in South Texas, from 2017 to 2021. Vertical zero line denotes no selection, values to the left represent avoidance while values to the right represent selection of that variable. Variables include percent land cover (PLAN), edge density (ED), patch density (PD), landscape shape index (LSI), and mean patch area (MPA) for three cover types (bare, herbaceous and woody cover), as well as probability of bobcat and coyote 2nd order use in the case of ocelot habitat selection.

**Figure 3:**
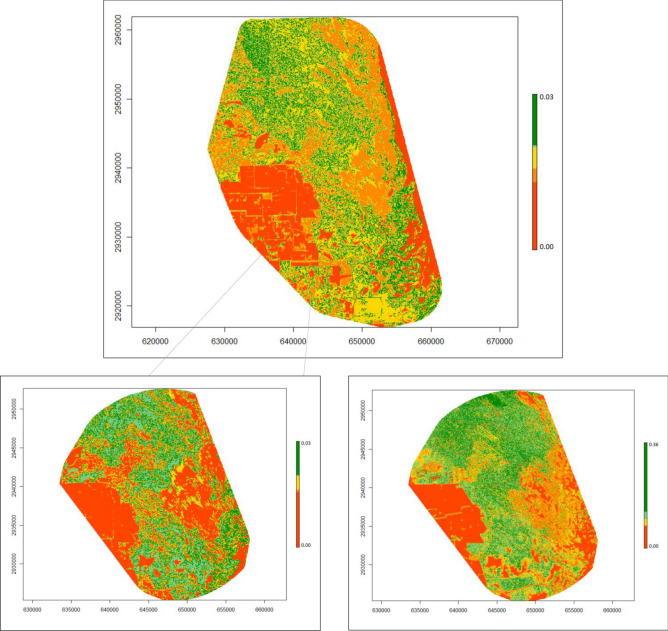
Heat maps displaying the probability of use of bobcats (*Lynx rufus*) in South Texas, USA from 2017 to 2021. The top panel shows 2nd order selection, the bottom panels compare 2nd order selection (left) to 3rd order selection (right) within the area used for 3rd. Colors are binned across five quantiles (from red to dark green), according to each set of values, to provide easier comparison across orders/species. For a measure of scale, axes are labeled in Universal Trans Mercator (UTM); every 1000 units represents 1 km. Figure was created in R Studio 1.2 (www.rstudio.com).

**Figure 4:**
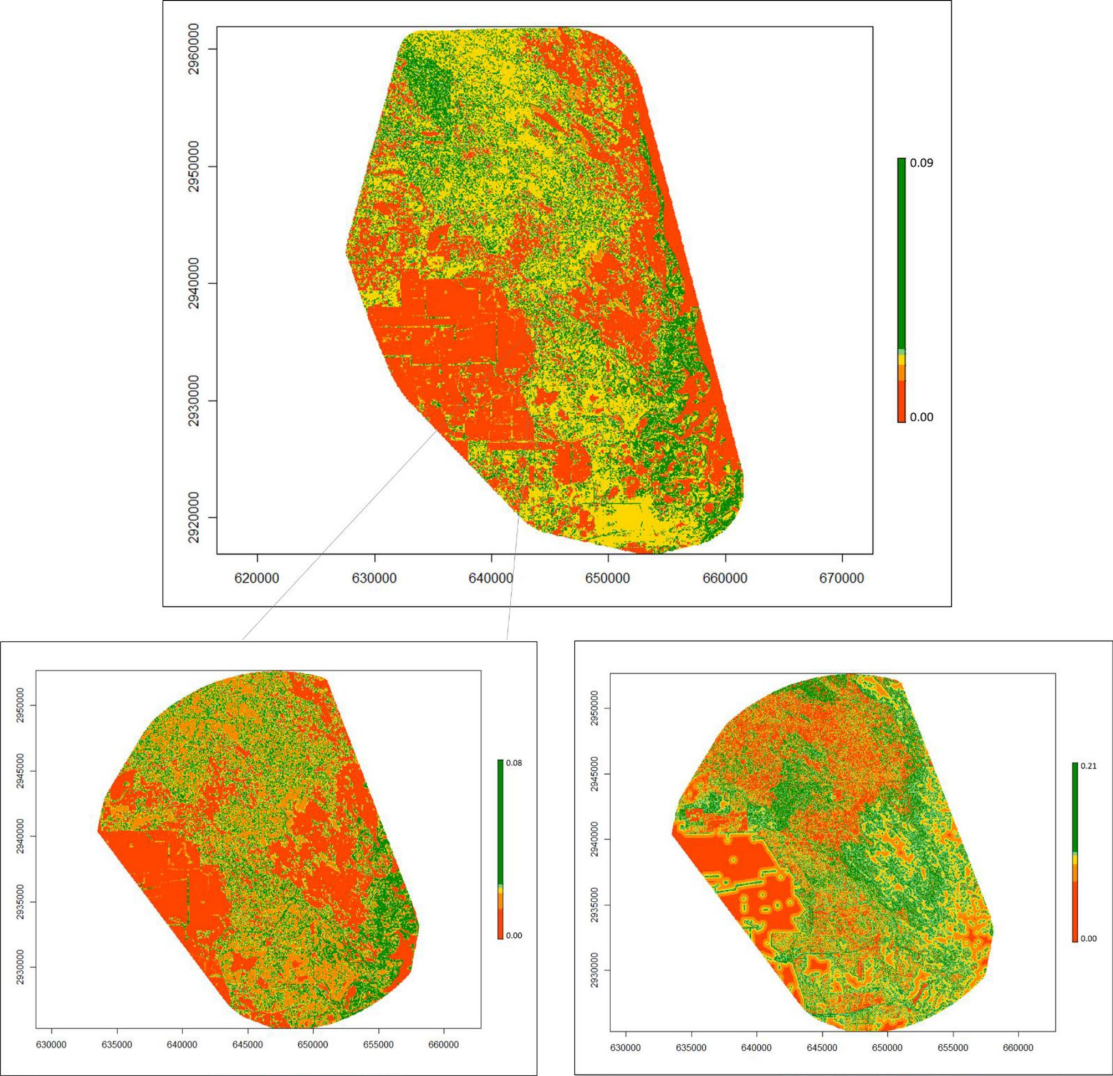
Heat maps displaying the probability of use of coyotes (*Canis latrans*) in South Texas, USA from 2017 to 2021. The top panel shows 2nd order selection, the bottom panels compare 2nd order selection (left) to 3rd order selection (right) within the area used for 3rd. Colors are binned across five quantiles (from red to dark green), according to each set of values, to provide easier comparison across orders/species. For a measure of scale, axes are labeled in Universal Trans Mercator (UTM); every 1000 units represents 1 km. Figure was created in R Studio 1.2 (www.rstudio.com).

**Figure 5:**
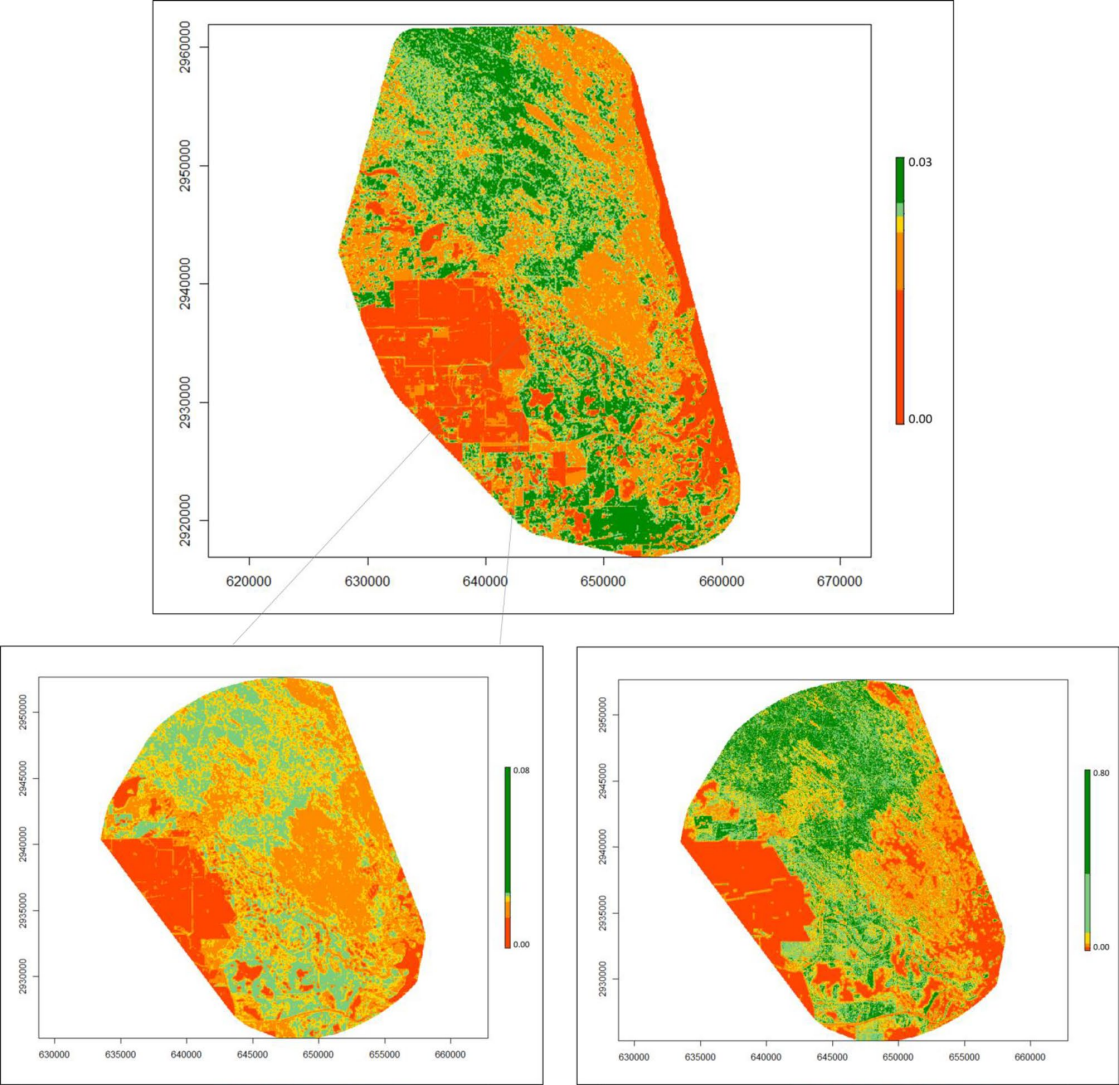
Heat maps displaying the probability of use by ocelots (*Leopardus pardalis*) in South Texas, USA from 2017 to 2021. The top panel shows 2nd order selection, the bottom panels compare 2nd order selection (left) to 3rd order selection (right) within the area used for 3rd. Colors are binned across five quantiles (from red to dark green), according to each set of values, to provide easier comparison across orders/species. For a measure of scale, axes are labeled in Universal Trans Mercator (UTM); every 1000 units represents 1 km. Figure was created in R Studio 1.2 (www.rstudio.com).

At the 2nd order, habitat selection of bobcats was best predicted by woody mean patch area (MPA), woody patch density (PD), herbaceous MPA, herbaceous edge density (ED), bare MPA, and bare ED (∆AIC_c_ > 2.0; Table 1, Figs. 2 & 3). Bobcats selected for larger patches of each cover type, such that odds of use increased by 18.4% (OR = 1.18, 95% CI [1.16, 1.21]), 13.1% (OR = 1.13, 95% CI [1.11, 1.15]) and 7.3% (OR = 1.07, 95% CI [1.06, 1.09]) for each standard deviation increase in size of woody, herbaceous, and bare ground respectively. Bobcats selected greater herbaceous ED and greater woody PD, such that a standard deviation increase in herbaceous edge and woody patch density increased odds of use by 4.0% (OR = 1.04, 95% CI [1.02, 1.06]) and 7.0% (OR = 1.07, 95% CI [1.05, 1.09]) respectively (Table 2). Our top model for habitat selection of coyotes included landscape shape index (LSI) of woody, herbaceous and bare (∆AIC_c_ > 2.0; Table 1, Figs. 2, 4). Coyotes selected for areas with larger values of LSI for each cover type, such that each standard deviation increase in LSI of woody, herbaceous and bare ground increased odds of use by 34.0% (OR = 1.34, 95% CI [1.32, 1.36]), 6.4% (1.06, 95% CI [1.05, 1.08]), and 14.0% (OR = 1.14, 95% CI [1.12, 1.15]; Table 2) respectively, showing a preference for fragmented lands (LSI begins at 1.0 for a single large patch and increases as fragmentation increases). The top model describing the habitat use of ocelots included PLAND of woody, herbaceous and bare cover, woody and bare ED, herbaceous PD, and the probability of use by bobcats and coyotes (∆AIC_c_ = 1.90; Table 1, Figs. 2, 5). Ocelots selected for greater PLAND of woody and herbaceous cover, such that a standard deviation increase in woody and herbaceous PLAND was associated with 12.4% (OR = 1.13, 95% CI [1.06, 1.20]) and 11.8% (OR = 1.12, 95% CI [1.08, 1.17]) increase in odds of use. Ocelots selected for lower ED of woody and bare ground, such that a standard deviation increase in in ED of woody and bare cover decreased odds of use by 3.8% (OR = 0.96, 95% CI [0.93, 1.00]) and 2.0% (OR = 0.98, 95% CI [0.96, 1.00]) respectively (Table 2). We observed selection for areas of higher probability of use by bobcats (OR = 1.13, 95% CI [1.07, 1.20]) while probability of coyote use had no effect (OR = 1.01, 95% CI [0.97, 1.05]).

**Table 1:** AICc model selection describing 2nd order selection of bobcats (*Lynx rufus*), coyotes (*Canis latrans*) and ocelots (*Leopardus pardalis*) in South Texas, USA from 2017 to 2021.

Model	AIC_c_	∆AIC_c_	Weight
Bobcats			
WoodyMPA + WoodyPD + HerbMPA + HerbED + BareMPA + BareED	257,379.9	0.00	1.00
WoodyPLAN + WoodyED + HerbPLAN + HerbPD + BarePLAN + BareED	257,407.5	27.59	0.00
WoodyPLAN + WoodyPD + HerbED + HerbMPA	257,516.5	136.58	0.00
WoodyPLAN + BarePLAN + HerbPLAN	257,519.1	139.25	0.00
WoodyED + WoodyMPA + HerbPD + HerbMPA	257,532.8	152.91	0.00
Coyotes			
WoodyLSI + HerbLSI + BareLSI	166,933.0	0.00	1.00
WoodyLSI + HerbED + BarePLAN	166,949.3	16.32	0.00
WoodyMPA + WoodyPD + HerbMPA + HerbED + BareMPA + BareED	166,965.9	32.93	0.00
WoodyPLAN + WoodyED + HerbPLAN + HerbPD + BarePLAN + BareED	167,030.2	97.21	0.00
BarePLAN + BareED + WoodyPLAN + WoodyED	167,070.1	137.12	0.00
Ocelots			
WoodyPLAN + WoodyED + HerbPLAN + HerbPD + BarePLAN + BareED + Bobcat2ndProb + Coyote2ndProb	314,280.1	0.00	0.539
WoodyPLAN + HerbMPA + Bobcat2ndProb + Coyote2ndProb	314,282.0	1.90	0.209
WoodyMPA + WoodyED + HerbMPA + HerbED + BareMPA + BareED + Bobcat2ndProb + Coyote2ndProb	314,283.1	3.01	0.119
WoodyPLAN + BarePLAN + HerbPLAN + Bobcat2ndProb + Coyote2ndProb	314,284.7	4.57	0.055
WoodyED + WoodyMPA + HerbPD + HerbMPA + Bobcat2ndProb + Coyote2ndProb	314,285.1	5.01	0.044

Top five models out of 12 candidate models are shown (10 in the case of ocelots). Variables include mean patch area (MPA; ha), landscape shape index (LSI [the ratio of the actual landscape edge length to the minimum possible edge length]), edge density (ED; m/ha), patch density (PD; patches/100 ha), and percent land cover (PLAND; %) for three cover types (bare, herbaceous and woody cover), and probability of bobcat and coyote 2nd order use in the case of ocelot habitat selection. All variables in the models were scaled and centered.

**Table 2:** Parameter estimates from the top model, according to AICc, describing 2nd habitat selection of bobcats (*Lynx rufus*), coyotes (*Canis latrans*) and ocelots (*Leopardus pardalis*) in South Texas USA from 2017 to 2021.

	Estimate	St. error	p-value
Bobcat			
Intercept	− 3.9267	0.0086	< 0.0001
WoodyMPA	0.1686	0.0110	< 0.0001
WoodyPD	0.0678	0.0096	< 0.0001
HerbMPA	0.1234	0.0103	< 0.0001
HerbED	0.0393	0.0081	< 0.0001
BareMPA	0.0701	0.0077	< 0.0001
BareED	0.0096	0.0074	0.194
Coyote			
Intercept	− 3.9403	0.0285	< 0.0001
WoodyLSI	0.2923	0.0086	< 0.0001
HerbLSI	0.0623	0.0081	< 0.0001
BareLSI	0.1306	0.0064	< 0.0001
Ocelots			
Intercept	− 3.9130	0.0216	< 0.0001
WoodyPLAN	0.1170	0.0319	0.0002
WoodyED	− 0.0385	0.0176	0.0290
HerbPLAN	0.1112	0.0233	< 0.0001
HerbPD	− 0.0052	0.0102	0.6057
BarePLAN	0.0080	0.0125	0.5214
BareED	− 0.0204	0.0100	0.0410
Bobcat2ndProb	0.1256	0.0291	< 0.0001
Coyote2ndProb	0.0069	0.0211	0.7443

Variables include percent land cover (PLAN; %), edge density (ED; m/ha), patch density (PD; patches/100 ha), landscape shape index (LSI), and mean patch area (MPA; ha) for three cover types (bare, herbaceous, and woody cover), as well as probability of bobcat and coyote 2nd order use in the case of ocelot habitat selection. All variables in the models were scaled and centered.

**Figure 6:**
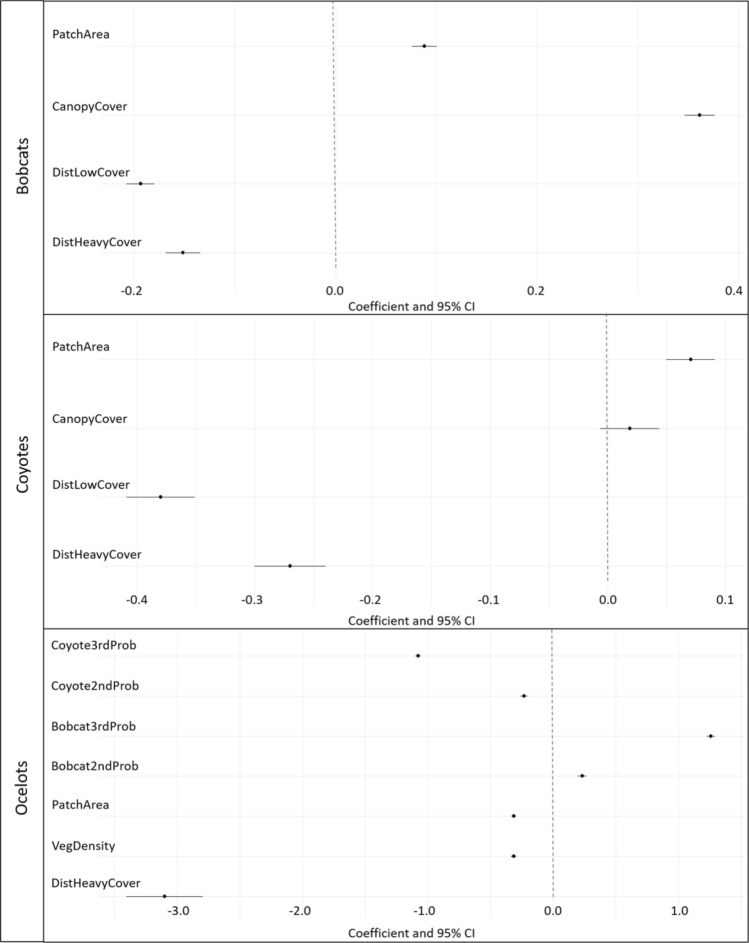
Model coefficients from most supported models describing the 3rd order habitat selection of bobcats (*Lynx rufus*), coyotes (*Canis latrans*) and ocelots (*Leopardus pardalis*) in South Texas, from 2017 to 2021. Vertical zero line denotes no selection, values to the left represent avoidance while values to the right represent selection of that variable. Variables include distance to dense cover (> 75% canopy cover), distance to low cover/open areas (< 25% canopy cover), patch area of dense cover, percent canopy cover and vegetation density, as well as probability of bobcat and coyote 2nd order use and probability of bobcat and coyote 3rd order use in the case of ocelot habitat selection.

**Figure 7:**
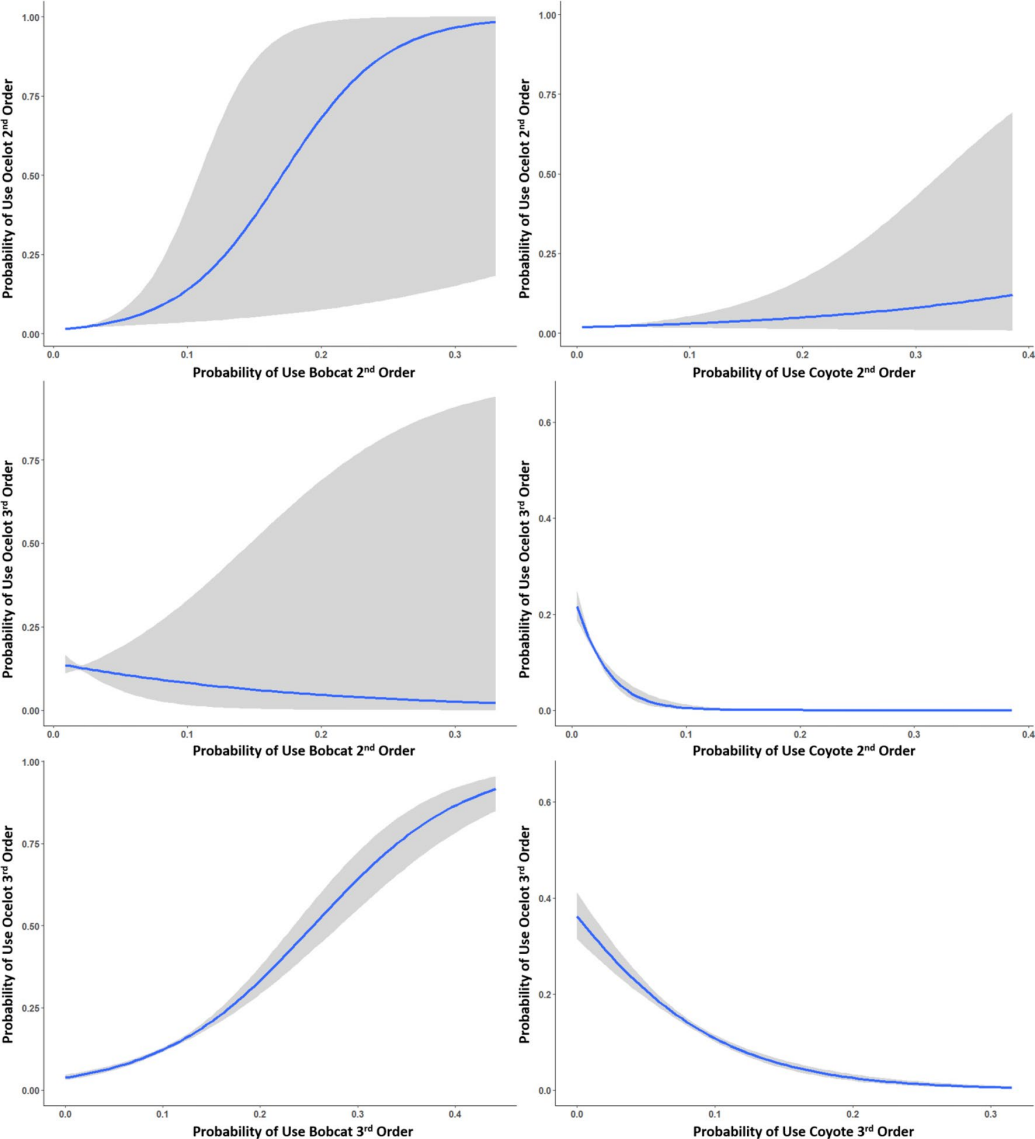
Predictive plots showing the probability of use by ocelots (*Leopardus pardalis*) in relation to probability of use by bobcats (*Lynx rufus*) and coyotes (*Canis latrans*), at the 2nd and 3rd order, in South Texas, USA from 2017 to 2021.

At the 3rd order, our top model of habitat selection of bobcats included distance to dense cover, distance to open areas, percent canopy cover, and patch area of dense cover (∆AIC_c_ > 2.0; Table 3, Fig. 6). Bobcats selected for areas closer to dense cover and open areas, such that each standard deviation increase in distance to dense cover and open areas decreased odds of use by 14.0% (OR = 0.86, 95% CI [0.84, 0.87]) and 17.6% (OR = 0.82, 95% CI [0.81, 0.84]) respectively. Bobcats selected larger patches of dense cover (OR = 1.09, 95% C.I. [1.08, 1.10]), and greater canopy cover (OR = 1.43, 95% CI [1.41, 1.46]; Table 4). Habitat selection of coyotes was also best predicted by distance to dense cover, distance to open areas, canopy cover, and patch area (∆AIC_c_ = 1.10; Table 3, Fig. 6). We elected not to average models with the second competing model as the predictor variables only differed by the substitution of vegetation density for canopy cover and these variables were highly correlated. Within their home range, coyotes selected areas closer to dense cover and open areas, such that a standard deviation increase in distance to dense cover and open areas was associated with a decrease in odds of use of 23.6% (OR = 0.76, 95% CI [0.74, 0.79]) and 31.6% (OR = 0.68, 95% CI [0.66, 0.70]) respectively, and selected larger patches of woody cover (odds ratio = 1.07, 95% CI [1.05, 1.10]; Table 4). Third order selection of ocelots was best predicted by distance to dense cover, patch area, vegetation density, probability of use by bobcats at the 2nd and 3rd order, and probability of use by coyotes at the 2nd and 3rd order (∆AIC_c_ > 2.0; Table 3, Fig. 6). Ocelots selected areas closer to dense cover, such that a standard deviation increase in distance to dense cover decreased odds of use by 95.5% (OR = 0.04, 95% CI [0.03, 0.06]), and selected lower vegetation density (odds ratio = 0.73, 95% CI [0.72,0.74]) and smaller patches of dense cover (OR = 0.73, 95% CI [0.72, 0.74]; Table 4). Ocelots selected for areas with higher probability of use by bobcats at the 2nd and 3rd order (OR = 1.26, 95% CI [1.22, 1.31] for 2nd order use and 3.53, 95% C.I. [3.42, 3.64] for 3rd order use; Fig. 7). Ocelots avoided areas used by coyotes at both the 2nd and 3rd order (OR = 0.79, 95% CI [0.77, 0.82] for 2nd order use and 0.34, 95% CI [0.33, 0.35] for 3rd order use).

**Table 3:** AICc model selection describing 3rd order selection of bobcats (*Lynx rufus*), coyotes (*Canis latrans*) and ocelots (*Leopardus pardalis*) in South Texas, USA from 2017 to 2021.

Model	AIC_c_	∆AIC_c_	Weight
**Bobcats**			
DistHeavy + DistLow + CanopyCover + PatchArea	342,027.9	0.00	1.00
DistLow + CanopyCover + PatchArea	342,385.9	358.02	0.00
DistHeavy + DistLow + VegDensity + PatchArea	342,616.6	588.63	0.00
CanopyCover + PatchArea + DistHeavy	342,851.1	823.15	0.00
DistLow + VegDensity + PatchArea	343,306.9	1278.99	0.00
**Coyotes**			
DistHeavy + DistLow + CanopyCover + PatchArea	121,740.6	0.00	0.635
DistHeavy + DistLow + VegDensity + PatchArea	121,741.7	1.10	0.365
DistHeavy + DistLow + PatchShape	121,781.5	40.90	0.000
DistHeavy + DistLow	121,783.3	42.68	0.000
DistLow + CanopyCover + PatchArea	122,184.4	443.80	0.000
**Ocelots**			
DistHeavyCover + VegDensity + PatchArea + Bobcat2ndProb + Bobcat3rdProb + Coyote2ndProb + Coyote3rdProb	188,437.8	0.00	1.00
DistHeavyCover + PatchArea + Bobcat2ndProb + Bobcat3rdProb + Coyote2ndProb + Coyote3rdProb	189,302.5	864.71	0.00
VegDensity + PatchArea + Bobcat2ndProb + Bobcat3rdProb + Coyote2ndProb + Coyote3rdProb	189,523.9	1086.10	0.00
DistHeavyCover + VegDensity + Bobcat2ndProb + Bobcat3rdProb + Coyote2ndProb + Coyote3rdProb	189,886.5	1448.67	0.00
PatchArea + Bobcat2ndProb + Bobcat3rdProb + Coyote2ndProb + Coyote3rdProb	190,055.6	1617.74	0.00

Top 5 models out of 12 candidate models are shown (8 in the case of ocelots). Variables include distance to dense cover (> 75% canopy cover; DistHeavyCover), distance to low cover/open areas (< 25% canopy cover, DistLowCover), patch area of dense cover (Patch Area, m^2^), percent canopy cover (CanopyCover) and vegetation density (Veg density; vegetation points/cell), as well as probability of bobcat and coyote 2nd order use and probability of bobcat and coyote 3rd order use in the case of ocelot habitat selection.All models included animal ID as a random effect. All variables in the models were scaled and centered.

**Table 4:** Parameter estimates from the top model, according to AICc, describing 3rd habitat selection of bobcats (*Lynx rufus*), coyotes (*Canis latrans*) and ocelots (*Leopardus pardalis*) in South Texas USA from 2017 to 2021.

	Estimate	St. error	p-value
**Bobcat**			
Intercept	− 2.2712	0.0692	< 0.0001
DistHeavyCover	− 0.1513	0.0088	< 0.0001
DistLowCover	− 0.1935	0.0071	< 0.0001
CanopyCover	0.3613	0.0077	< 0.0001
Patch Area	0.0882	0.0064	< 0.0001
**Coyote**			
Intercept	− 2.3472	0.0118	< 0.0001
DistHeavyCover	− -0.2699	0.0155	< 0.0001
DistLowCover	− 0.3799	0.0148	< 0.0001
CanopyCover	0.0186	0.0129	0.149
Patch area	0.0703	0.0105	< 0.0001
**Ocelots**			
Intercept	− 3.4437	0.1830	< 0.0001
DistHeavyCover	− 3.0987	0.1559	< 0.0001
Veg density	− 0.3143	0.0108	< 0.0001
Patch area	− 0.3117	0.0087	< 0.0001
Bobcat2ndProb	0.2333	0.0173	< 0.0001
Bobcat3rdProb	1.2604	0.0163	< 0.0001
Coyote2ndProb	− 0.2297	0.0143	< 0.0001
Coyote3rdProb	− 1.0765	0.0111	< 0.0001

Variables include distance to dense cover (> 75% canopy cover; DistHeavyCover), distance to low cover/open areas (< 25% canopy cover, DistLowCover), patch area of dense cover (Patch Area, m^2^), percent canopy cover (CanopyCover) and vegetation density (Veg Density; vegetation points/cell), as well as probability of bobcat and coyote 2nd order use and probability of bobcat and coyote 3rd order use in the case of ocelot habitat selection. All variables in the models were standardized.

## Discussion

As a species of conservation concern, understanding the effects of competitor species on the habitat selection of ocelots is vital to management. We compared habitat selection of ocelots, bobcats and coyotes and examined if ocelots spatially avoided these competitors across two orders of selection. By leveraging landscape-level and LiDAR data we were able to take an unprecedented examination of fine-scale habitat selection by these sympatric carnivores, providing new insights and confirming observations in prior studies. At the broader level (2nd order), we observed overlap between species and no avoidance of competitors by ocelots. At a finer scale (3rd order), we observed fine-scale habitat partitioning that may reflect interspecific niche partitioning or be a result of competition for spatial resources. Further, we observed avoidance of coyotes by ocelots at the 3rd order, further emphasizing niche partitioning between species and revealing scale-dependent patterns in avoidance of competitor species.

At a broader scale, bobcats positioned their home ranges in areas with larger patches of woody, bare and herbaceous cover types suggesting a preference for heterogeneous landscapes that were comprised of all three cover types. Bobcats selected greater herbaceous edge density and higher woody patch density, suggesting a preference for vegetation cover and interspersion of cover types, supported by our results of 3rd order habitat selection. Within their home ranges, bobcats selected areas near dense vegetation cover and open areas, further supporting a generalist pattern and selection for edges, consistent with past literature suggesting generalist habitat use^59,60^. Our results suggest selection for multiple cover types and edges across both scales examined. Prior assessments of habitat selection by bobcats have shown differences in selection across scale and stress the importance of comparing patterns at both a broad and fine scale^51,61–63^. Bobcats in southeastern United States selected croplands at a fine scale but selected pine and hardwood habitat at a broader scale^63,64^. Gene flow in a population of bobcats was influenced differently by cover type across varying spatial scale^51^. Bobcats avoided high elevations and heavy snow at a broad scale and selected for forest, shrub and wetland at a finer scale^62^. These prior studies show scale-dependent differences in habitat selection, however, we show similar patterns at both the 2nd and 3rd order wherein bobcats selected for edges and an interspersion of multiple cover types.

Coyotes selected a broad range of cover types with fragmented patches and areas close to edges across scales; however, we observed selection for vegetated areas at the 2nd order and use of open herbaceous areas at the 3rd order (Fig. 4). In the western United States, coyotes showed consistent selection across the 2nd and 3rd order, showing a preference for early successional vegetation^65^. Chamberlain^66^ similarly found selection for a variety of cover types; however, they observed seasonal differences across scale but found consistent selection for pine stands in the winter. At a broad scale, coyotes selected open vegetation types and recently burned forests while at finer scales they avoided dense vegetation and paved roads^67^. We found similar patterns between coyotes and bobcats, showing selection for all three cover types with heavy interspersion, suggesting a high degree of overlap between home ranges of bobcats and coyotes. This is consistent with long-term camera trapping data of the study area, which showed these species were up to six times more likely to co-occur in the same areas^32^. This high extent of overlap suggests these two species are not avoiding each other at the broad scale, either due to a lack of competition or due to partitioning the landscape at a finer spatial or temporal scale^31^. We did find some evidence of partitioning at the finer scale. While both species selected areas closer to dense vegetation cover and open areas, bobcats selected greater canopy cover while coyotes showed no relationship and showed much greater use of open areas than bobcats and ocelots (Figs. 3, 4 and 5). Past literature on competition between coyotes and bobcats is equivocal. Some studies have found a high degree of overlap in habitat selection and home ranges while others have not, however, in most cases co-existence is attributed to fine-scale habitat partitioning between species^38,39,68,69^, similar to our findings on bobcats and coyotes.

When positioning their home range, ocelots selected areas for greater woody and herbaceous cover, with larger woody patches and less bare ground consistent with recent studies that used landscape metrics to describe ocelot selection or use. Ocelots selected large, contiguous patches^20,21,27,30,70^ with lower edge densities^30,71^ across their geographic range. Larger areas of herbaceous vegetation within home ranges may also help facilitate movement when patrolling territory^31^ or establishing den sites^50^. Our results also contrast Jackson et al.^72^ which showed selection for fragmented areas (i.e. large patches with higher shape indices) in a smaller subpopulation of ocelots located 30 km south of our study area. At the 2nd order, ocelots selected for areas of greater probability of use by bobcats and showed no effect associated with probability of coyotes, suggesting ocelots were not avoiding either species at the broader scale. Similarly, Lombardi et al.^32^ found that ocelots were 10–12 times more likely to occur in areas occupied by coyotes and bobcats based on long-term camera trapping data. This overlap in resources may be driven by behavioral-mediated co-occurrence of activity patterns and finer scale partitioning of cover^31^ which allowed each species to coexist even when there may be competition when selecting areas for home ranges. Horne et al.^26^ similarly found overlap in the positioning of home ranges by bobcats and ocelots and a prior study comparing home range placement of ocelots with two other sympatric carnivores, ring-tailed coatis (*Nasua nasua*) and crab-eating foxes (*Cerdocyon thous*), similarly found overlap in home range placement among species^73^.

At the 3rd order, ocelots selected areas closer to dense vegetation cover, consistent across scales, which support results from prior literature and similar to bobcats and coyotes, suggesting a possibility of interspecific competition for dense vegetation communities. Selection between ocelots and coyotes suggested less overlap, similar to the comparison between bobcats and coyotes. We observed a negative relationship between ocelots and patch area and vegetation density, which would partially contradict prior understanding^70^ however, these differences may also be a result of variation in individual selection or selection of different canopy characteristics^74^. Within their home ranges, ocelots selected areas with a higher probability of use by bobcats at the 2nd and 3rd order, suggesting they were neither avoiding bobcat home ranges nor areas used by bobcats at a finer scale. While we saw no avoidance of coyotes at the 2nd order, at the 3rd order ocelots avoided both coyote home ranges and areas used by coyotes within their home range showing differences in habitat selection and competitor avoidance across scales. Reasons why ocelots are directly avoiding coyotes only at the third order and not 2nd order are unclear; avoidance may be the result of mutual avoidance or interspecific competition, wherein the presence of coyotes is directly influencing habitat selection of ocelots. These interactions may also be an artifact of niche partitioning or temporal segregation between species, reflecting real differences in ecological niches. Further research would be required to support one hypothesis over the other and would require examination of avoidance by coyotes or a comparison of habitat use by ocelots before and after coyote removal to identify an explanation.

Comparisons of habitat selection between ocelots and bobcats in South Texas showed overlap in position of home ranges, consistent with our results, and found evidence of fine-scale habitat partitioning as a means of coexistence^26^, although our results showed very similar selection between bobcats and ocelots at the fine-scale. Dietary overlap between these two species is similar but enough variation exist to reduce some degree of competition^42^. To our knowledge, only two studies have examined coexistence between ocelots, bobcats, and coyotes in a single study and they examined presence using camera traps^32^, and hidden Markov movement models to examine behavioral differences in resource selection^31^, as opposed to multiscale habitat selection as in the case of our study. Further, prior understanding of the habitat selection of ocelots have also come from either camera traps^16,20,73,75^ or telemetry^19,25–27,72^. Our study benefitted from the use of high-frequency GPS data collected from all three species examined, providing a more in-depth understanding of habitat selection and potential avoidance between species.

Our study is the first to examine avoidance of functionally similar carnivores by ocelots; however, coexistence among carnivores has been examined in other portions of the ocelot’s range. In South America, where bobcats and coyotes are absent, ocelots coexist with larger, more dominant predators such pumas (*Puma concolor*) and jaguar (*Panthera onca*) and were positively associated with the presence of these larger predators^35–37^, suggesting no negative top-down effects from these larger felids on ocelots. Within these regions, coexistence of ocelots with jaguars and pumas was attributed to temporal and spatial partitioning^35,76,77^. While larger predators did not negatively influence the presence, the diet of ocelots shifted to larger prey species in the absence of more dominant predators^78^, suggesting competition influences the realized niche of ocelots; a process that may be occurring within our study area as a result of competition from coyotes. Alternatively, as jaguars did exist within this study historically, predator release may be acting on coyotes, in the absence of jaguars, allowing them to act as more dominant predators. Ocelots did not avoid sympatric felids in Brazil but avoided domestic dogs^79^, similar to the avoidance of a sympatric canid and lack of avoidance of other felids that we observed in our study, although niche partitioning was higher among morphologically similar carnivores in Argentina^36^. Conversely, ocelots had a negative influence on the habitat use and activity of smaller felids and other mesocarnivores^35,36,80^, suggesting the potential for a similar dynamic within our study region wherein ocelots have a negative influence on coyotes through competition, potentially resulting in the habitat partitioning we observed in our study. Coexistence among sympatric carnivores has been attributed to spatial heterogeneity^81,82^ and we similarly provide evidence of spatial partitioning within a heterogeneous landscape as an explanation for coexistence among ocelots, bobcats and coyotes. Our results expand upon previous literature on the coexistence of ocelots with dominant and subordinate predators by describing patterns of avoidance with predators of a similar trophic level.

In addition to the inferences drawn about habitat selection of these three carnivores and patterns of coexistence and avoidance by ocelots, our study provides another example of the importance of scale in ecological research. Habitat selection is a dynamic process that occurs across multiple scales simultaneously^1^. We therefore recommend a thorough consideration of scale be undertaken when formulating ecological hypotheses. Had we examined only 2nd order selection, we would have found no patterns of avoidance of sympatric carnivores by ocelots, and conversely had we only examined the 3rd order we might have falsely concluded that ocelots avoid coyotes at any scale (i.e. transmutability). In addition to scale-effects, some differences we observed may have been related to a small sample size. The concept of scale has become increasingly important in studies examining habitat selection. Recent studies have considered selection across multiple scales and shown differences across scales^3,13,14,83^. A multiscale approach has been applied to ocelots twice before^26,73^, however, the use of high-frequency GPS data in our analysis allows for a deeper level of inference over radio telemetry and emphasizes greater selection for herbaceous cover at the broad scale and scale dependent habitat partitioning and avoidance of competitor species. We recommend that, whenever possible, studies consider habitat selection across multiple scales to identify scale-dependent trends.

We provide the first comparison of habitat selection of ocelots, bobcats and coyotes and compare selection across two orders (2nd and 3rd) and examine avoidance of competitor species by the endangered ocelot. We show that fine-scale habitat partitioning is occurring to facilitate coexistence between species, whereby bobcats and coyotes showed selection for a wide range of cover types and use of open areas by coyotes while ocelots were strongly tied to dense (woody and herbaceous) vegetation. We found no avoidance of competitor species by ocelots at the 2nd order, suggesting similar habitat requirements among species at the broader scale. At the 3rd order, however, we detected avoidance of coyotes but not bobcats, showing differing patterns of avoidance across scale and species. Avoidance of coyotes may reflect a competition for space between species or may simply reflect differences in ecological niche between species, thereby reducing competition. The high degree of overlap with bobcats, particularly at the fine-scale, may alternatively be a source of interspecific competition for ocelots, as these species may compete for optimal patches. Our results provide a guideline for landscape management and emphasize the importance of woody and herbaceous cover at the landscape level and patches of dense vegetation at the home-range level to sustain populations of ocelots. Further, in considering areas for reintroduction of ocelots, we provide an initial analysis to examine the impact of competitor species on ocelots. Future research may be conducted to experimentally exclude competitors to compare habitat selection of ocelots or, alternatively, examine interspecific avoidance by bobcats or coyotes to better elucidate directionality of potential competition. As a species of conservation concern, understanding the habitat selection of ocelots and the role of competitor species in influencing habitat selection is of vital importance to conserving ocelots in South Texas.

## Data Availability

We are unable to make GPS location data publicly available due to the sensitive nature of ocelots as an endangered species, however, with proper permitting and approval, data can be made available to interested parties. For such a request, please contact the corresponding author. We are able to provide R code associated with the analysis.
